# Role of AQP4 in ameliorating heat stress-induced cellular injury in a cell line model through active heat acclimation

**DOI:** 10.3389/fnhum.2026.1830585

**Published:** 2026-06-16

**Authors:** Xin Li, Chuhan Xiang, Yan Zhao, Qing Liu, Shuhua Cao, Fan Xu, Ying Sun, Lizhen Ma

**Affiliations:** 1 Department of Emergency, Third Medical Center of Chinese PLA General Hospital, Beijing, China; 2 Department of Cardiology, The 71st Group Army Hospital of CPLA Army, Xuzhou, China; 3 Beijing University of Chinese Medicine, Beijing, China; 4 Academy of Military Medical Sciences, Beijing, China; 5 Department of Oncology, The Fifth Medical Center of Chinese PLA General Hospital, Beijing, China

**Keywords:** apoptosis, aquaporin-4, heat acclimatization, heat shock proteins, heat stress, heat stroke

## Abstract

**Introduction:**

Heat stress (HS) can progress to heat stroke, a life-threatening condition. Aquaporin-4 (AQP4) has been implicated in HS-induced brain injury, but its role in heat acclimation (HA)-mediated protection remains unclear. This study investigated whether HA ameliorates HS-induced brain damage through AQP4.

**Methods:**

9L/lacZ cells were randomly assigned to four groups: Control (37 °C, 5% CO₂), HA (39 °C, 5% CO₂, 2 h/day for 6 days), HS (43 °C, 5% CO₂, 2 h), and HA+HS (HA pretreatment followed by HS). In addition, cells in the Control and HS groups were treated with the selective AQP4 inhibitor TGN-020 (0.1 μmol/mL, 2 h before HS). The expression of HSP70, HSP90, and AQP4 was measured by western blotting; AQP4 mRNA levels were assessed by RT-PCR. Cell proliferation viability was evaluated by CCK-8 assay, and apoptosis was detected by flow cytometry.

**Results:**

Notably, AQP4 expression (RT-PCR and Western blot) was lower in the HA+HS group than in the HS group. Flow cytometry revealed that both HA+HS and TGN-020 treatment reduced apoptosis compared with HS alone.

**Discussion:**

These findings indicate that HA attenuates HS-induced injury by downregulating AQP4 expression, and that AQP4 inhibition mimics this protective effect. AQP4 represents a potential therapeutic target for heat stroke.

## 1 Introduction

Heat stroke is a severe clinical syndrome that is characterized by hyperthermia and central nervous system (CNS) damage, often accompanied by multiple organ failure (Liu et al., 2020). The onset of HS is characterized by its rapid progression, with a high mortality rate and the potential for survivors to experience prolonged neurological sequelae (Ma et al., 2026; Bouchama et al., 2022). The pathological basis of heat stroke is an excessive heat stress (HS) response. The heat stress response is primarily associated with heat accumulation in the body, dehydration, and electrolyte disturbances (Wang et al., 2022).

Aquaporin-4 (AQP4), the predominant water channel in the mammalian CNS, is densely expressed in astrocytic end-feet, glial limiting membranes, and ependymal lining, where it facilitates bidirectional water flux and maintains brain water homeostasis (Sachdeva et al., 2023; Tait et al., 2008; Badaut et al., 2002; Sobue et al., 2006; Szczygielski et al., 2021). Under physiological conditions, AQP4 is essential for normal cerebrospinal fluid circulation and glymphatic clearance. Nevertheless, accumulating evidence indicates that AQP4 plays a dichotomous role in pathological states. While AQP4 deficiency can impair the resolution of vasogenic edema, its overexpression accelerates cytotoxic swelling by facilitating excessive water influx during acute insults such as ischemia, trauma, and HS (Wang et al., 2022; Sandhya et al., 2023; Yang et al., 2008). Our previous animal study demonstrated that AQP4 expression was markedly upregulated in the brain tissue of rats subjected to exertional heat stroke, concomitant with increased brain water content (Li et al., 2023; Du et al., 2017). These findings suggest that aberrant AQP4 upregulation contributes to HS-induced cytotoxic brain edema, positioning AQP4 as a potential therapeutic target for mitigating CNS damage in heat stroke.

Heat acclimation (HA) is defined as a process by which the body gradually adapts to high temperatures and improves heat tolerance through repeated exposure to heat (Price, 2015). It has been demonstrated that HA can enhance the body’s thermal tolerance through a variety of mechanisms, including lowering the metabolic rate, increasing the cardiovascular reserve, and the capacity of the evaporative cooling system (Zurawlew et al., 2016). Research has demonstrated that HA can significantly ameliorate multi-system organ damage caused by HS; however, the precise mechanism remains to be fully elucidated (Chapman et al., 2021; Lee et al., 2016).

In this study, we established an *in vitro* cellular model of HA and HS using 9L/lacZ rat glioma cells to dissect the role of AQP4 in HA-mediated cytoprotection. Based on our prior *in vivo* observations, we hypothesized that (i) HA attenuates HS-induced cellular injury and apoptosis by downregulating the pathological upregulation of AQP4; (ii) pharmacological inhibition of AQP4 by TGN-020 would mimic the protective effect of HA against HS-induced apoptosis. We assessed AQP4 expression at both mRNA and protein levels, evaluated cell proliferation and apoptosis, and examined the functional consequences of selective AQP4 inhibition. Our findings aim to elucidate whether AQP4 acts as a mechanistic mediator of HA-induced tolerance to HS and to provide a theoretical framework for developing fine-tuned, AQP4-targeted interventions for heat stroke prevention and treatment.

## 2 Materials and methods

### 2.1 Cell culture and treatments

The 9L/lacZ cells are purchased from the Shanghai Institute of Cell Biology, Chinese Academy of Sciences. Cells are grown in high glucose DMEM medium 84%+high quality fetal bovine serum 15%+bispecific antibodies [penicillin and streptomycin (1%)]. Cells were maintained at 37 °C in a humidified atmosphere containing 5% CO₂. For heat acclimation (HA), cells were exposed to 40 °C, 5% CO₂ for 2 h per day for six consecutive days (Patton et al., 2018). For heat stress (HS), cells were incubated at 43 °C, 5% CO₂ for 2 h. The HA+HS group received HA pretreatment (40 °C, 2 h/day for 6 days) followed immediately by HS exposure (43 °C, 2 h). The control group was maintained at 37 °C throughout the experiment.

The selective AQP4 inhibitor TGN-020 (CAS 51987-99-6, MedChemExpress, USA) was dissolved in dimethyl sulfoxide (DMSO) to a stock concentration of 10 mmol/L and further diluted in culture medium to a final concentration of 0.1 μmol/mL (100 μmol/L). For TGN-020 intervention, cells were pre-treated with TGN-020 at 37 °C, 5% CO₂ for 2 h before subsequent HS or control incubation. The control+TGN-020 group was treated with TGN-020 alone at 37 °C for 2 h, while the HS+TGN-020 group received TGN-020 pre-treatment followed by HS (43 °C, 2 h). All experiments were performed in triplicate.

### 2.2 CCK-8 assays

The assessment of cell viability was performed using the Cell Counting Kit-8 (CCK-8) from Dojindo, Japan, following the provided protocol. Cells from various experimental conditions were quantified and diluted to a density of 1 × 10^5^ cells/ML. These were then distributed into a 96-well plate at a volume of 100 μL/well, with each condition replicated three times. The plate was incubated under standard conditions (37 °C, 5% CO_2_) until the cells reached the desired confluence. At this point, 10 μL of CCK-8 reagent was introduced into each well, and the plate was returned to the incubator for an additional 3 h. Post-incubation, the optical density at 450 nm was measured using a microplate reader (Bio-Tek, USA). Measurements included blank controls (containing only culture medium and CCK-8) and untreated cell controls (cells with culture medium and CCK-8) to ensure accurate baseline readings. Untreated control cells were used as the reference for 100% viability.

### 2.3 Real-time quantitative PCR

Total RNA was isolated using Trizol reagent (Invitrogen, CA, USA), followed by quantification and quality assessment of the extracted RNA. RNA concentration and purity were determined spectrophotometrically (NanoDrop 3,000, Thermo Fisher, USA). The reaction mixture was prepared using the TB Green Premix Ex Taq kit from Takara. Quantitative PCR was performed on the QuantStudio 7 Flex real-time fluorescence quantitative PCR system to analyze the expression levels of the target genes. The cycling conditions were: 95 °C for 30 s, followed by 40 cycles of 95 °C for 5 s and 60 °C for 34 s. The sequences of the primers used are listed below. AQP4: F: 5′-TTAACTGGGGTGGCTCAGAG-3′, R: 5′-GCGATGCTGATC TTTCGTGT-3′. β-actin: F: 5′-CACGATGGAGGGGCCGGACTCAT C-3, R: 5′-TAAAGACCTCTATGCCAACACAGT-3′. Relative gene expression was calculated using the 2^−ΔΔCt^ method, with β-actin as the internal reference.

### 2.4 Western blot

Cells were lysed in RIPA buffer (Beyotime, China) supplemented with protease and phosphatase inhibitors (Roche, Switzerland). Protein concentration was determined using a BCA assay kit (Thermo Fisher). The extracted proteins were then resolved via 10% sodium dodecyl sulfate-polyacrylamide gel electrophoresis (SDS-PAGE) and subsequently transferred onto nitrocellulose membranes (Millipore, USA). The membranes were blocked with 5% non-fat milk in Tris-buffered saline containing 0.1% Tween-20 for 1 h at room temperature, then incubated overnight at 4 °C with primary antibodies targeting HSP70 (1:1000, Abcam, ab278579), HSP90 (1:1000, Abcam, ab13495), AQP4 (1:1000, Abcam, ab46182), and GAPDH (1:5000, Proteintech, 60004-1-Ig). After three washes with TBST, membranes were incubated with horseradish peroxidase (HRP)-conjugated secondary antibodies (goat anti-rabbit IgG, 1:5000) for 1 h at room temperature. Protein bands were visualized using enhanced chemiluminescence (ECL) reagent (Millipore) and imaged with a Bio-Rad system. Band intensities were quantified using ImageJ software, and expression levels were normalized to GAPDH.

### 2.5 Flow cytometry analysis of cell apoptosis

The apoptosis assay was conducted following the instructions provided with the Annexin V-FITC apoptosis detection kit (Invitrogen). Approximately 1 × 10^6^ cells were harvested, rinsed with ice-cold PBS, and then re-suspended in binding buffer supplemented with 5 μL of Annexin V-FITC. The cells were incubated in the dark at room temperature for 10 min, after which the buffer was discarded via centrifugation. The cell pellet was subsequently re-suspended in reaction buffer containing 1 μL of PI (100 μg/mL). Samples were analyzed within BD FACSCanto™ II flow cytometer (BD Biosciences, USA). At least 10,000 events were recorded per sample, and each experiment was repeated three times (Xia et al., 2026).

### 2.6 Statistical analysis

All data are presented as mean ± standard deviation (SD) from at least three independent experiments. Comparisons between two groups were performed using unpaired Student’s *t*-test, and multiple group comparisons were carried out by one-way analysis of variance (ANOVA) followed by Tukey’s *post-hoc* test. Statistical significance was set at *p* < 0.05. All analyses were conducted using GraphPad Prism 9.0 (GraphPad Software, USA).

## 3 Results

### 3.1 HA cell models successfully established

**Figure 1:**
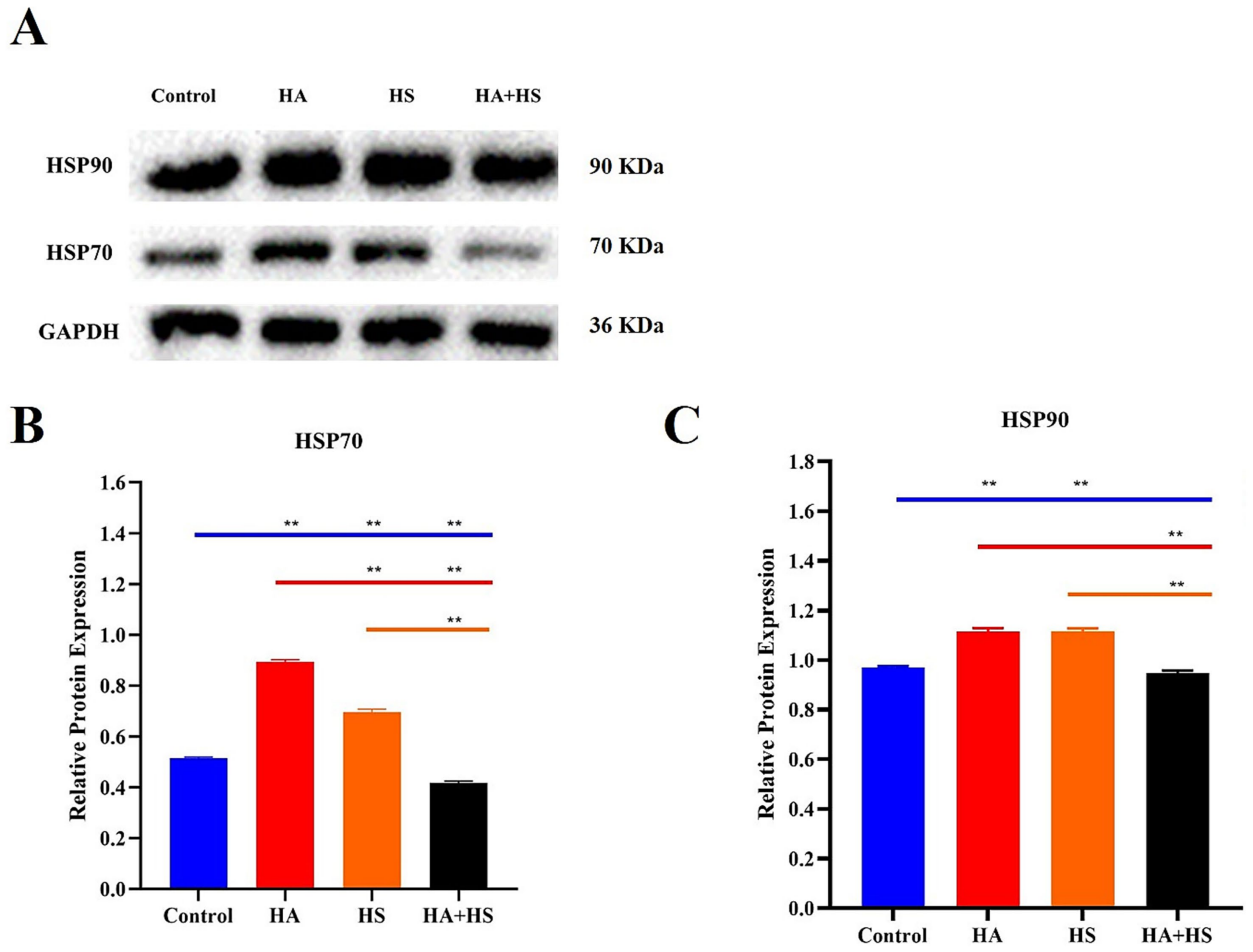
Evaluation of HA and HS cell model in 9L/lacZ cells. **(A)** Representative Western blot results of HSP70 and HSP90 in different groups; **(B)** Western blot analysis of HSP70 expression levels in different groups; **(C)** Western blot analysis of HSP90 expression levels in different groups, data are mean ± SD. *p < 0.05, **p < 0.01.

The 9L/lacZ cells were subjected to heat acclimation (HA), heat stress (HS), or their combination (HA+HS). After treatment, cell lysates were collected and analyzed by Western blotting to detect the protein expression levels of HSP70 and HSP90 (Figure 1A). As shown in Figures 1B,C, compared with the Control group, the expression of both HSP70 and HSP90 was significantly increased in the HA, HS, and HA+HS groups, with the highest expression observed in the HA+HS combination group. These results indicate that the cellular models of heat acclimation and heat stress have been successfully established.

### 3.2 HA enhances the proliferation of HS cells

**Figure 2:**
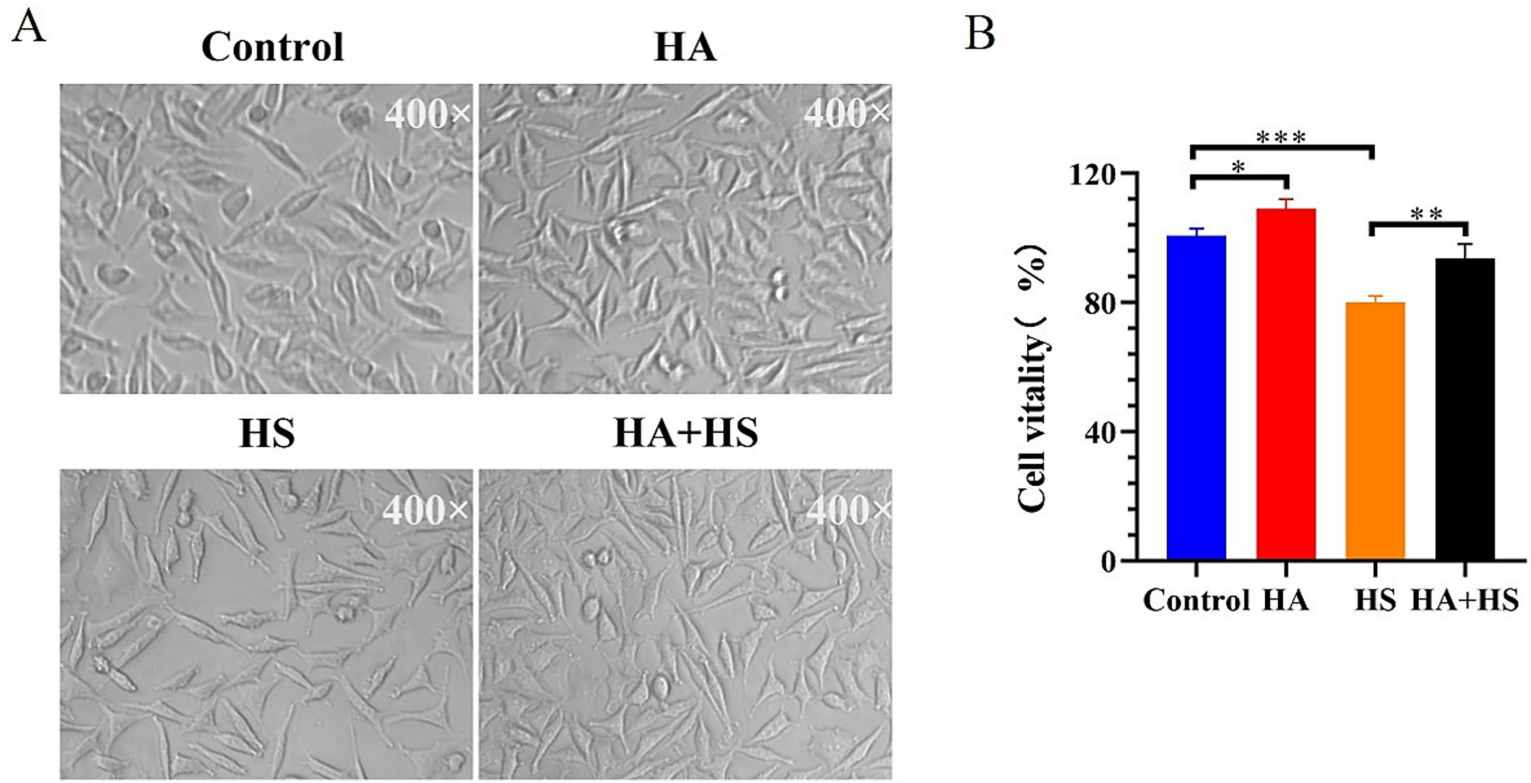
HA enhances cell proliferation after HS. **(A)** Light microscopy images of cell growth in each group; **(B)** cell viability assessed using the CCK-8 assay, data are mean ± SD. *p < 0.05, **p < 0.01, ***p < 0.001.

Morphological observation revealed inadequate cell adhesion and increased floating cells in the HS group, whereas the HA+HS group displayed improved cell integrity and growth status compared with HS alone (Figure 2A). CCK-8 was conducted to assess the effect of the HA treatment on the cell survival rate. The results demonstrated that the HA group exhibited a higher cell survival rate in comparison to the control group (*p* < 0.001). Conversely, HA pretreatment significantly preserved proliferative capacity, with the HA+HS group exhibiting substantially higher viability than the HS group (*p* < 0.01; Figure 2B). Notably, the HA group alone showed a modest but significant increase in cell survival compared with Control (*p* < 0.05), indicating that sub-lethal thermal preconditioning does not compromise basal proliferative function.

### 3.3 Differential regulation of AQP4 by HA at transcriptional and translational levels

**Figure 3:**
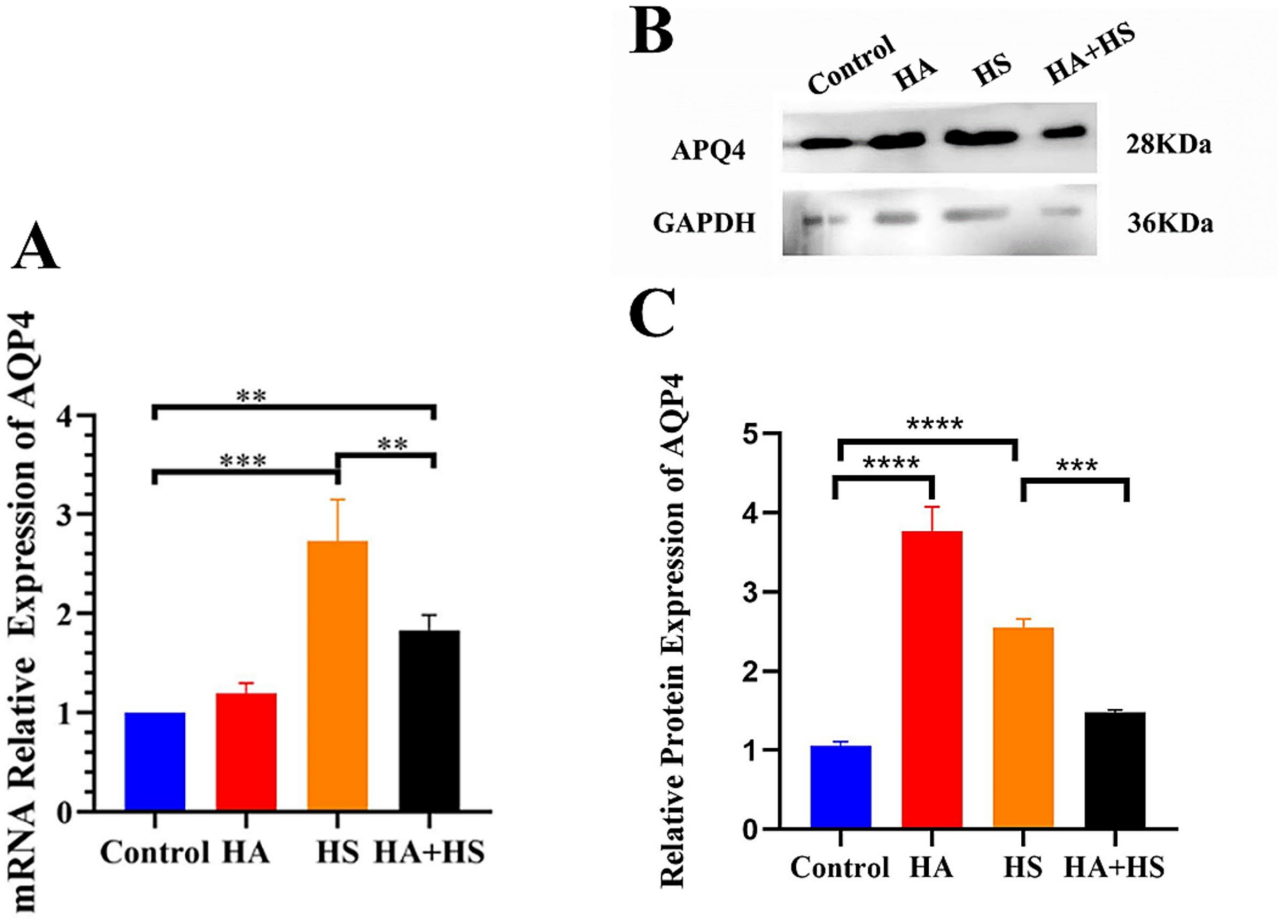
AQP4 expression is downregulated by heat acclimation following HS. **(A)** RT-PCR analysis of AQP4 mRNA expression; **(B)** representative western blot results of AQP4 in different groups; **(C)** Western blot analysis of AQP4 expression levels in different groups, data are mean ± SD. *p < 0.05, **p < 0.01, ***p < 0.001, ****p < 0.0001.

To investigate the molecular mechanism underlying HA-mediated cytoprotection, we analyzed AQP4 expression following HS challenge. Quantitative RT-PCR revealed that HS robustly upregulated AQP4 mRNA expression compared with Control (*p* < 0.01; Figure 3A). Consistent with our previous proteomic findings (Li et al., 2023). AQP4 mRNA levels in the HA+HS group were significantly attenuated relative to the HS group (*p* < 0.01), though remaining moderately above baseline. Surprisingly, the HA group alone displayed AQP4 mRNA levels comparable to or only marginally elevated above Control, yet significantly lower than both HS and HA+HS groups. Strikingly, Western blot analysis uncovered a marked dissociation between mRNA and protein expression patterns (Figures 3B,C). While HS significantly elevated AQP4 protein abundance relative to Control (*p* < 0.0001), the HA group exhibited the most pronounced upregulation, with AQP4 protein levels significantly exceeding those in Control (*p* < 0.0001), HS (*p* < 0.001), and HA+HS (*p* < 0.0001) groups. Meanwhile, the HA+HS group showed significantly reduced AQP4 protein expression compared with HS alone (*p* < 0.001), approaching baseline levels. This uncoupling of mRNA and protein expression in the HA group suggests that repetitive sub-lethal heat exposure may enhance AQP4 protein stability or translational efficiency through post-transcriptional mechanisms, independent of transcriptional activation.

### 3.4 AQP4 modulation correlates with apoptotic responses during HS

**Figure 4:**
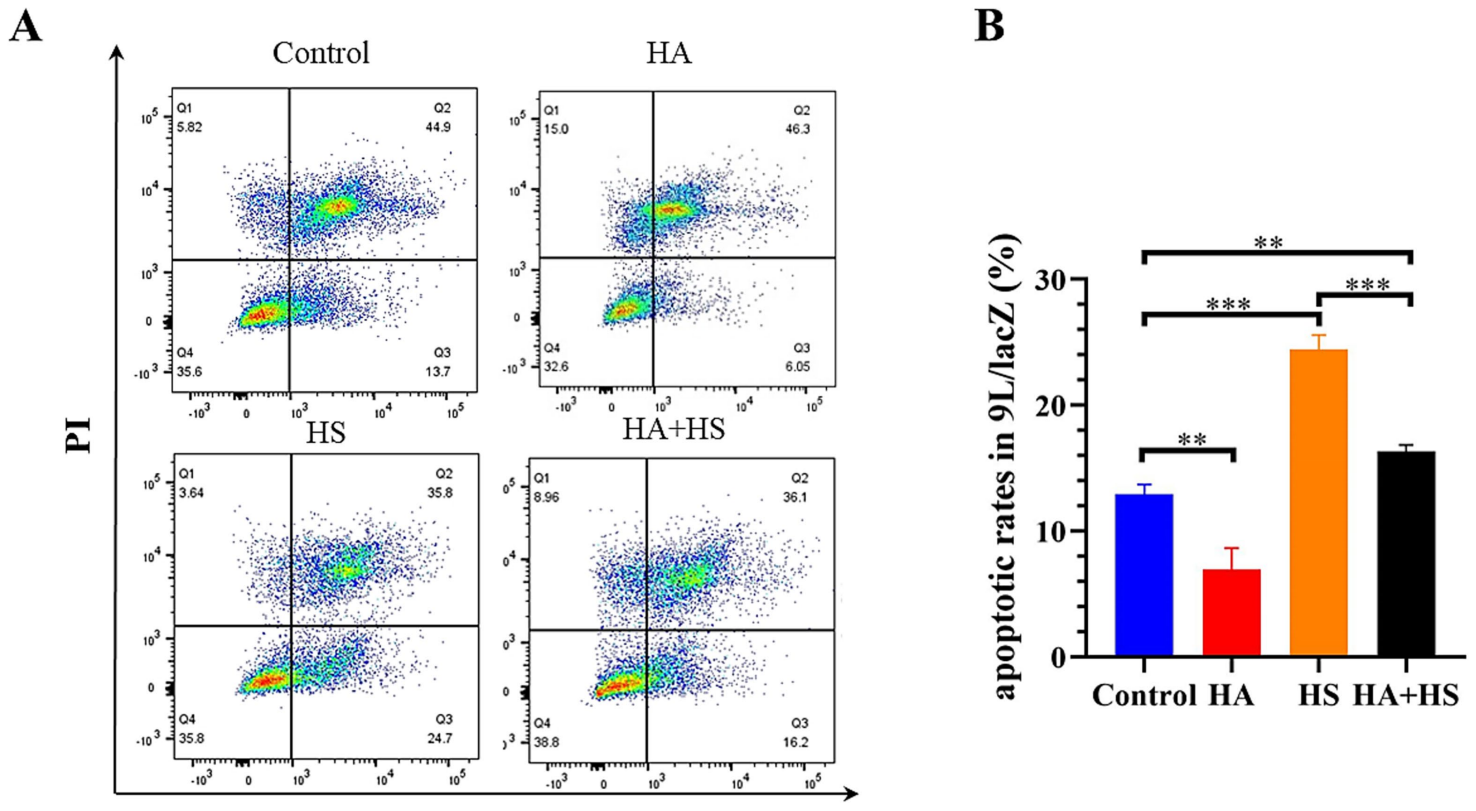
HA reduces HS-induced apoptosis in 9L/lacZ cells. **(A)** Representative dot plots results of each group detected by flow cytometry; **(B)** flow cytometry analysis of Annexin V-FITC/PI double staining, data are mean ± SD. **p < 0.01, ***p < 0.001.

Flow cytometric analysis of Annexin V-FITC/PI staining revealed that HS dramatically increased the apoptotic rate compared with Control (*p* < 0.001), whereas HA pretreatment significantly mitigated HS-induced apoptosis (HA+ HS vs. HS, *p* < 0.01; Figure 4), correlating with the downregulation of AQP4 expression observed in the HA+HS group.

**Figure 5:**
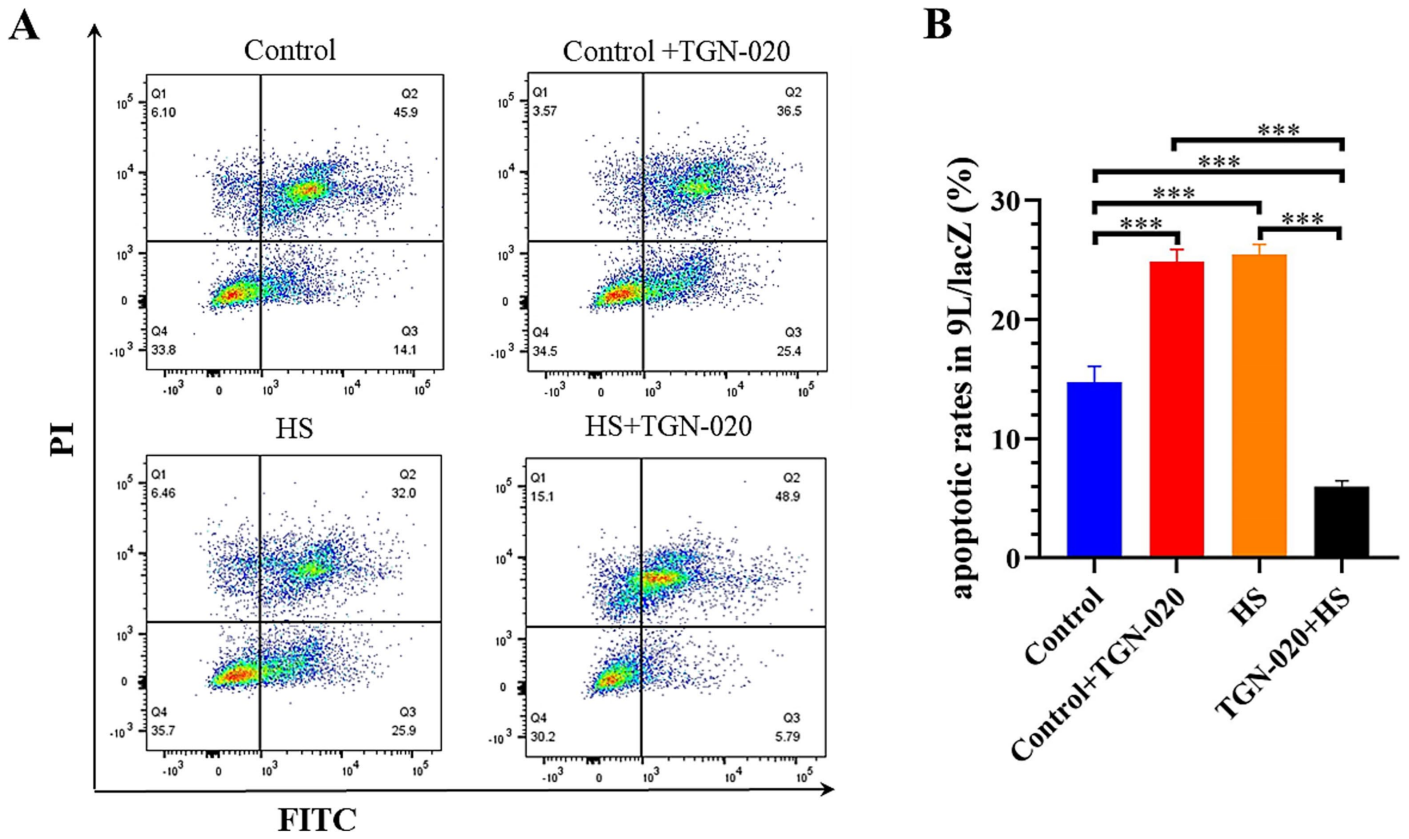
Pharmacological inhibition of AQP4 with TGN-020 mimics the anti-apoptotic effect of HA under HS. **(A)** Representative dot plots results of each group detected by flow cytometry; **(B)** flow cytometry analysis of Annexin V-FITC/PI double staining, data are mean ± SD. **p < 0.01, ***p < 0.001.

To functionally validate the role of AQP4, we employed the selective AQP4 inhibitor TGN-020. As shown in Figure 5, pharmacological inhibition of AQP4 significantly attenuated HS-induced apoptosis (HS+TGN-020 vs. HS, *p* < 0.001), closely phenocopying the protective effect of HA preconditioning. However, under non-stress conditions, TGN-020 treatment paradoxically increased apoptosis in the Control+TGN-020 group compared with Control (*p* < 0.01). These results indicate that AQP4 exerts a context-dependent dual function: while AQP4 suppression protects against HS-induced cellular injury, its inhibition under physiological conditions disrupts basal homeostasis and promotes apoptosis, consistent with the elevated AQP4 protein levels observed in the HA group.

## 4 Discussion

In this study, we investigated the regulatory effects of HA on AQP4 expression and its effects on apoptosis and proliferation through an *in vitro* cellular model. The fact that HA can attenuate HS-induced brain injury by reducing AQP4 expression provides a theoretical basis for the development of therapeutic strategies based on AQP4 regulation. For example, pharmacological inhibition of AQP4 expression or the development of molecular interventions that can mimic the effects of heat exposure are expected to be new directions in the prevention and treatment of HS. This mechanistic exploration provides a new molecular target for the treatment of HS, i.e., attenuating HS-induced brain injury by regulating AQP4 expression, with important clinical translational potential. Our key findings show that HA provides robust protection against heat stress-induced cellular damage, as evidenced by enhanced cell viability, reduced apoptosis and, critically, a reduced induction of AQP4 expression in response to lethal heat exposure. The protective phenotype of HA was mimicked in HS-exposed cells by pharmacological inhibition of AQP4 with TGN-020, underscoring AQP4 as a pivotal effector in HA-mediated cytoprotection. Interestingly, HA alone induced a distinctive AQP4 expression profile characterized by increased protein abundance without a corresponding increase in mRNA, suggesting that thermal adaptation reprograms AQP4 regulation at the post-transcriptional level. Taken together, these observations support a model in which HA remodels cellular proteostasis to prevent the pathological surge in AQP4 that drives HS-induced apoptosis and proliferation impairment.

### 4.1 HA attenuates HS-induced injury and reduces AQP4 expression

Consistent with our previous proteomic findings in an exertional heat stroke rat model (Li et al., 2023), the present study demonstrated that HA pretreatment significantly lowered AQP4 mRNA and protein levels in 9L/lacZ cells following HS, compared with the HS group. This reduction was accompanied by improved cell viability and decreased apoptosis. Given that AQP4 is the predominant water channel in the central nervous system and facilitates pathological water influx during cytotoxic edema (Sachdeva et al., 2023; Yang et al., 2017), our results support the notion that HA alleviates HS-induced cellular injury by suppressing excessive AQP4 expression. The successful establishment of the HA model was confirmed by elevated HSP70 and HSP90 levels, which are well-established molecular markers of thermal adaptation (Price, 2015; Zurawlew et al., 2016).

### 4.2 Baseline reprogramming of AQP4 during HA

A novel observation in this study is the marked discordance between AQP4 mRNA and protein expression in the HA group. While HA cells exhibited significantly higher AQP4 protein levels compared to control cells, their mRNA levels remained only marginally elevated. This transcription–translation decoupling implies that repeated sub lethal heat exposure enhances AQP4 protein stability or translational efficiency rather than driving sustained transcriptional activation. Such post-transcriptional accumulation may represent an adaptive proteostatic reserve that primes cells for subsequent severe stress. As a membrane channel protein, AQP4 typically possesses a longer half-life than its transcript; thus, reduced proteasomal or lysosomal degradation during the adaptation phase, potentially mediated by heat shock protein chaperoning (e.g., HSP70/HSP90), and could account for protein accumulation without proportional mRNA increases. Alternatively, HA may suppress specific microRNAs or RNA-binding proteins that otherwise constrain AQP4 translation, thereby allowing protein synthesis to proceed despite stable transcript levels. This baseline reprogramming appears to fundamentally alter the cellular transcriptional response to lethal HS. In naïve cells exposed directly to HS, AQP4 was robustly induced at both mRNA and protein levels, likely reflecting a maladaptive stress response that exacerbates cytotoxic edema and ionic dysregulation. In contrast, HA-pretreated cells subjected to HS (HA+HS group) displayed significantly attenuated AQP4 induction compared with the HS group. We hypothesize that the pre-existing AQP4 protein pool in HA cells, combined with heat-induced epigenetic or transcription factor modifications at the AQP4 locus, prevents the excessive transcriptional surge observed in naïve HS cells. Consequently, HA+HS cells maintain AQP4 expression within a physiological window that is sufficient for basal water homeostasis but insufficient to trigger the catastrophic swelling and apoptotic cascades characteristic of HS injury.

### 4.3 The dualistic role of AQP4 in physiological versus pathological thermal stress

Our findings with the selective AQP4 inhibitor TGN-020 provide compelling evidence for the context-dependent, dualistic nature of AQP4 in thermal stress responses. In control cells, TGN-020 administration significantly increased apoptosis, indicating that basal AQP4 expression is essential for maintaining cellular viability, likely by facilitating physiological water and solute transport and preventing osmotic stress. Conversely, in HS-exposed cells, TGN-020 markedly reduced apoptosis, mirroring the protective effect observed in the HA+HS group. These divergent outcomes suggest that AQP4 exerts a Janus-faced role: it is cytoprotective at physiological expression levels but becomes deleterious when pathologically upregulated during severe thermal stress. Under physiological conditions, a basal level of AQP4 expression might be required for normal cellular homeostasis, possibly through its involvement in water and ion balance, cell volume regulation, or even subtle signaling functions (Tani et al., 2008). In contrast, under pathological HS conditions, HS-induced upregulation of AQP4 becomes detrimental, promoting excessive water influx, cellular swelling, and subsequent apoptosis. This biphasic, context-dependent role of AQP4 has also been noted in other disease models. For example, Yang et al. (2008) reported that AQP4 overexpression in transgenic mice accelerated cytotoxic brain swelling, while AQP4 deficiency under certain conditions can be detrimental. Our results extend this concept to the setting of HS and HA.

### 4.4 Potential signaling pathways and future directions

The mechanistic link between HA, AQP4 modulation, and apoptosis attenuation remains to be fully elucidated, but existing literature points toward the PI3K/Akt signaling cascade as a plausible integrator. Multiple studies have demonstrated that aquaporins interact with PI3K/Akt/mTOR pathways to regulate cell survival, migration, and stress responses. In the context of thermal injury, Akt phosphorylation could serve sequential functions: (i) during the HA phase, promoting AQP4 protein stabilization and anti-apoptotic signaling to establish a pro-survival phenotype; and (ii) upon HS challenge, suppressing the transcriptional hyper activation of AQP4 by modulating transcription factor activity (e.g., NF-κB or HIF-1α) or chromatin accessibility at the AQP4 promoter.

Although the present study did not directly investigate downstream signaling, the observation that HA and TGN-020 both reduce apoptosis suggests that AQP4 may interact with pro-apoptotic pathways. Previous studies have implicated the PI3K/Akt signaling cascade in AQP4 regulation (Chen et al., 2021; Zhu et al., 2015). In spinal cord injury, modulation of the PTEN/AKT pathway reduced AQP4 expression and alleviated oedema (Chen et al., 2021). In adipocytes and hepatocytes, insulin and leptin control aquaporin expression via the PI3K/Akt/mTOR axis (Rodríguez et al., 2011). Given that Akt is a well-known anti-apoptotic kinase, it is plausible that HA-induced downregulation of AQP4 may relieve AQP4-mediated suppression of the PI3K/Akt pathway, thereby tipping the balance toward cell survival. Future studies using pathway-specific inhibitors or AQP4 gene silencing are warranted to test this hypothesis directly.

## 5 Conclusion

In summary, our *in vitro* findings demonstrate that HA protects 9L/lacZ cells against HS-induced injury by downregulating AQP4 expression, and that pharmacological inhibition of AQP4 mimics this protective effect under HS conditions. However, the pro-apoptotic effect of AQP4 inhibition in norm thermic cells highlights the need for context-dependent therapeutic strategies. These results identify AQP4 as a promising but delicate therapeutic target for heat stroke, and provide a cellular basis for further *in vivo* validation.

### 5.1 Limitation

The present study elucidates the mechanism by which HA mitigates HS-induced brain injury by modulating AQP4 expression through an in vitro cellular model, thus identifying a novel molecular target for the treatment of HS. The results suggest that AQP4 plays a pivotal role in the pathophysiological process of HS, and the regulation of AQP4 expression through pharmacological intervention or the mimicking of the effects of HA is anticipated to emerge as a strategy for the prevention and treatment of HS. However, the study has some limitations, and further in-depth exploration of the mechanism of action of AQP4 and validation of its therapeutic potential in animal models and clinical studies are needed in the future.

1. Although cellular experiments have provided important clues to the mechanism of action of AQP4, HS is a complex multi-system disease, and its pathophysiological process *in vivo* may involve more factors. Therefore, future animal models are needed to further validate the role of AQP4 in HS and to explore its expression and function in different tissues and organs.
2. Although the hypothesis that AQP4 may be involved in the PI3K/Akt signaling pathway has been proposed, direct evidence for a molecular mechanism is lacking. Future studies could consider gene knockdown, overexpression or signaling pathway inhibitors to delve into the interaction of AQP4 with this pathway and its specific function in HS.
3. Although the AQP4 inhibitor TGN-020 showed some protective effects in *in vitro* experiments, its safety and efficacy for in vivo application have not been verified. More preclinical studies are needed in the future to evaluate the potential of AQP4 inhibitors as therapeutic agents for HS.
4. Despite the conclusions drawn from this study, it is important to acknowledge the inherent limitations of the 9L/lacZ cell model used here. Expanding the repertoire of cell models in future studies will be necessary.

## Data Availability

The datasets presented in this study can be found in online repositories. The names of the repository/repositories and accession number(s) can be found in the article/supplementary material.

## References

[ref1] Badaut J. Lasbennes F. Magistretti P. J. Regli L. (2002). Aquaporins in brain: distribution, physiology, and pathophysiology. J. Cereb. Blood Flow Metab. 22, 367–378. doi: 10.1097/00004647-200204000-00001, 11919508

[ref2] Bouchama A. Abuyassin B. Lehe C. Laitano O. Jay O. O’Connor F. G. . (2022). Classic and exertional heatstroke. Nat. Rev. Dis. Primers 8:8. doi: 10.1038/s41572-021-00334-6, 35115565

[ref3] Chapman C. L. Johnson B. D. Parker M. D. Hostler D. Pryor R. R. Schlader Z. (2021). Kidney physiology and pathophysiology during heat stress and the modification by exercise, dehydration, heat acclimation and aging. Temperature (Austin) 8, 108–159. doi: 10.1080/23328940.2020.1826841, 33997113 PMC8098077

[ref4] Chen J. Zhang C. Li S. Li Z. Lai X. Xia Q. (2021). Exosomes derived from nerve stem cells loaded with FTY720 promote the recovery after spinal cord injury in rats by PTEN/AKT signal pathway. J. Immunol. Res. 2021, 1–13. doi: 10.1155/2021/8100298, 34337080 PMC8294984

[ref5] Du Y. Xu J. T. Jin H. N. Zhao R. Zhao D. Du S. H. . (2017). Increased cerebral expressions of MMPS, CLDN5, OCLN, ZO1 and AQPS are associated with brain edema following fatal heat stroke. Sci. Rep. 7:1691. doi: 10.1038/s41598-017-01923-w, 28490769 PMC5431794

[ref6] Lee B. J. Miller A. James R. S. Thake C. D. (2016). Cross acclimation between heat and hypoxia: heat acclimation improves cellular tolerance and exercise performance in acute Normobaric hypoxia. Front. Physiol. 7:78. doi: 10.3389/fphys.2016.00078, 27014080 PMC4781846

[ref7] Li X. Xv F. Ma L. Z. Xing L. Zhao J. B. Zhi W. J. . (2023). Acquired heat acclimation in rats subjected to physical exercise under environmental heat stress alleviates brain injury caused by exertional heat stroke. Brain Res. 1811:148393. doi: 10.1016/j.brainres.2023.148393, 37150340

[ref8] Liu S. Y. Song J. C. Mao H. D. Zhao J.-B. Song Q. (2020). Expert consensus on the diagnosis and treatment of heat stroke in China. Mil. Med. Res. 7:1. doi: 10.1186/s40779-019-0229-2, 31928528 PMC6956553

[ref9] Ma Q. Dong J. Zhang X. Yang R. Wei Y. (2026). Sheng Mai san regulating the oxidative stress and mitochondrial damage to alleviate liver injury in heat stress rats. Animals (Basel) 16:1391. doi: 10.3390/ani16091391, 42121810 PMC13163120

[ref10] Patton M. G. Gillum T. L. Szymanski M. C. Gould L. M. Lauterbach C. J. Vaughan R. A. . (2018). Heat acclimation increases inflammatory and apoptotic responses to subsequent LPS challenge in C2C12 myotubes. Cell Stress Chaperones 23, 1117–1128. doi: 10.1007/s12192-018-0923-0, 29907924 PMC6111074

[ref11] Price M. J. (2015). Preparation of Paralympic athletes; environmental concerns and heat acclimation. Front. Physiol. 6:415. doi: 10.3389/fphys.2015.00415, 26834641 PMC4712300

[ref12] Rodríguez A. Catalan V. Gomez-Ambrosi J. García-Navarro S. Rotellar F. Valentí V. . (2011). Insulin- and leptin-mediated control of aquaglyceroporins in human adipocytes and hepatocytes is mediated via the PI3K/Akt/mTOR signaling cascade. J. Clin. Endocrinol. Metab. 96, E586–E597. doi: 10.1210/jc.2010-1408, 21289260

[ref13] Sachdeva R. Priyadarshini P. Gupta S. (2023). Aquaporins display a diversity in their substrates. J. Membr. Biol. 256, 1–23. doi: 10.1007/s00232-022-00257-7, 35986775

[ref14] Sandhya P. Akaishi T. Fujihara K. Aoki M. (2023). A novel association of osmotic demyelination in Sjogren’s syndrome prompts revisiting role of aquaporins in CNS demyelinating diseases: a literature review. Mult. Scler. Relat. Disord. 69:104466. doi: 10.1016/j.msard.2022.104466, 36584554

[ref15] Sobue K. Asai K. Katsuya H. (2006). Aquaporin water channels in the brain and molecular mechanisms of brain edema. Nihon Rinsho 64, 1181–1189. 16768129

[ref16] Szczygielski J. Kopańska M. Wysocka A. Oertel J. (2021). Cerebral microcirculation, perivascular unit, and glymphatic system: role of aquaporin-4 as the gatekeeper for water homeostasis. Front. Neurol. 12:767470. doi: 10.3389/fneur.2021.767470, 34966347 PMC8710539

[ref17] Tait M. J. Saadoun S. Bell B. A. Papadopoulos M. C. (2008). Water movements in the brain: role of aquaporins. Trends Neurosci. 31, 37–43. doi: 10.1016/j.tins.2007.11.003, 18054802

[ref18] Tani K. Hiroaki Y. Fujiyoshi Y. (2008). Aquaporin-4. Rinsho Shinkeigaku 48, 941–944. doi: 10.5692/clinicalneurol.48.941, 19198125

[ref19] Wang Y. Huang C. Guo Q. Chu H. (2022). Aquaporin-4 and cognitive disorders. Aging Dis. 13, 61–72. doi: 10.14336/AD.2021.0731, 35111362 PMC8782559

[ref20] Xia A. Li X. Chen X. Quan J. Wan J. Zheng C. . (2026). *Mycobacterium tuberculosis* Rv0927c inhibits the proliferation and promotes the intrinsic apoptosis of alveolar epithelial cells through targeting mitochondrial TUFM. Front. Immunol. 17:1720489. doi: 10.3389/fimmu.2026.1720489, 41929492 PMC13038895

[ref21] Yang M. Li Z. Zhao Y. Zhou F. Zhang Y. Gao J. . (2017). Outcome and risk factors associated with extent of central nervous system injury due to exertional heat stroke. Medicine 96:e8417. doi: 10.1097/MD.0000000000008417, 29095276 PMC5682795

[ref22] Yang B. Zador Z. Verkman A. S. (2008). Glial cell aquaporin-4 overexpression in transgenic mice accelerates cytotoxic brain swelling. J. Biol. Chem. 283, 15280–15286. doi: 10.1074/jbc.M801425200, 18375385 PMC2397463

[ref23] Zhu C. Jiang Z. Bazer F. W. Johnson G. A. Burghardt R. C. Wu G. (2015). Aquaporins in the female reproductive system of mammals. Front. Biosci. (Landmark Ed) 20, 838–871. doi: 10.2741/4341, 25553483

[ref24] Zurawlew M. J. Walsh N. P. Fortes M. B. Potter C. (2016). Post-exercise hot water immersion induces heat acclimation and improves endurance exercise performance in the heat. Scand. J. Med. Sci. Sports 26, 745–754. doi: 10.1111/sms.12638, 26661992

